# Targeted Treatment of Yaws With Household Contact Tracing: How Much Do We Miss?

**DOI:** 10.1093/aje/kwx305

**Published:** 2017-11-13

**Authors:** Louise Dyson, Michael Marks, Oliver M Crook, Oliver Sokana, Anthony W Solomon, Alex Bishop, David C W Mabey, T Déirdre Hollingsworth

**Affiliations:** 1 Mathematics Institute, University of Warwick, Coventry, United Kingdom; 2 School of Life Sciences, University of Warwick, Coventry, United Kingdom; 3 Clinical Research Department, Faculty of Infectious and Tropical Diseases, London School of Hygiene and Tropical Medicine, London, United Kingdom; 4 Ministry of Health and Medical Services, Honiara, Solomon Islands; 5 Hospital for Tropical Diseases, University College London Hospitals NHS Trust, London, United Kingdom

**Keywords:** contact tracing, household modeling, mass drug administration, modeling, *Treponema pallidum pertenue*, total community treatment, total targeted treatment, yaws

## Abstract

Yaws is a disabling bacterial infection found primarily in warm and humid tropical areas. The World Health Organization strategy mandates an initial round of total community treatment (TCT) with single-dose azithromycin followed either by further TCT or active case-finding and treatment of cases and their contacts (the Morges strategy). We sought to investigate the effectiveness of the Morges strategy. We employed a stochastic household model to study the transmission of infection using data collected from a pre-TCT survey conducted in the Solomon Islands. We used this model to assess the proportion of asymptomatic infections that occurred in households without active cases. This analysis indicated that targeted treatment of cases and their household contacts would miss a large fraction of asymptomatic infections (65%–100%). This fraction was actually higher at lower prevalences. Even assuming that all active cases and their households were successfully treated, our analysis demonstrated that at all prevalences present in the data set, up to 90% of (active and asymptomatic) infections would not be treated under household-based contact tracing. Mapping was undertaken as part of the study “Epidemiology of Yaws in the Solomon Islands and the Impact of a Trachoma Control Programme,” in September–October 2013.

## BIOLOGICAL BACKGROUND

Yaws, caused by *Treponema pallidum pertenue*, is targeted for eradication by 2020 (1). In 2012 the World Health Organization launched the Morges strategy for yaws eradication. This strategy is based on an initial round of total community treatment (TCT, often referred to for other diseases as mass drug administration) with azithromycin. The initial round of TCT is followed by total targeted treatment (TTT), consisting of case-finding surveys, with treatment of identified cases and their contacts (2). A number of studies have now demonstrated that implementation of this strategy can significantly reduce the prevalence of yaws at the community level (3, 4).

A particular challenge for yaws eradication is the occurrence of asymptomatic infection (1). In these cases, individuals are asymptomatic but have serological evidence of infection. In surveys it has been noted that the ratio of asymptomatic to clinical cases may be as high as 6–10:1 (5, 6). Relapse from asymptomatic infection to infectious yaws, which may occur for up to 10 years, therefore serves as a potential source of reintroduction of infection into the community (7, 8). Because these individuals are asymptomatic, clinical case detection is insufficient to identify them and guide treatment decisions. The occurrence of asymptomatic infection was a major driver behind the decision to include an initial round of mass treatment in the yaws eradication strategy in order to maximize treatment coverage of asymptomatic, infected individuals.

There are limited data to inform the optimal mass treatment strategy for yaws eradication. In particular there are no empirical data to guide the number of rounds or coverage of mass treatment required or to assess the optimal strategy for contact tracing and treatment (6). The epidemiology of yaws transmission remains poorly understood. Currently there are limited data on the relationship of asymptomatic, infected cases to active cases within households, schools, and communities, as well as how this changes before and after mass treatment. An improved understanding of these relationships is vital to determining the optimal approach that should be taken to the TTT phase of yaws eradication activities. For the purposes of TTT, the Morges strategy loosely defines a contact as a person who has close and frequent contact with the infected person. Contacts, for the purpose of yaws eradication, are members of the household, classmates, or close playmates as identified by the contact (2). Whether this strategy results in adequate coverage of asymptomatic, infected contacts of active cases is unknown.

In this study we used data from a pre-TCT household yaws survey conducted in the Solomon Islands to build a model of within- and between-household yaws transmission. We use this model to assess the coverage of active and asymptomatic cases that would be achieved if a household definition of contacts was applied.

## MODELING BACKGROUND

To assess the relative importance of infection within the household to infection in the general population, we employed a household model. Household models take account of the increased risk of transmission between individuals sharing a home by explicitly including different infection rates within the home and outside. These models vary in complexity according to the level of detail required to accurately model infection transmission and to answer the questions considered. The most complex models investigate disease transmission on highly resolved social networks (9). However gathering data on the network of contacts present within a village, in addition to disease status, is rarely achievable. Household models instead provide a tradeoff between level of detail and the availability of data, by using measurable household structures as a surrogate for the most important elements of a full transmission network. In addition, household-level treatment strategies may be more easily implemented than full contact tracing. Theoretical treatments have been undertaken for the susceptible-infectious-recovered models, deriving the final size of an epidemic (10). More recently Kinyanjui et al. (11) investigated the information content of household-stratified data, with the aim of designing studies to collect data to calibrate household models. Household models have been used to analyze influenza epidemics (12) and determine vaccination strategies for future influenza pandemics (13–15). Techniques for quantifying the impact of such an influenza pandemic have also been examined using data that could be gathered at the household level, early in a pandemic (16). Household-level targeting strategies have also been investigated for trachoma using a susceptible-infectious-susceptible model of infection (17), leading to the conclusion that the transmission rate within households is higher than that within the community.

## METHODS

### Data collection

Data were collected as part of a combined yaws and trachoma community-based, cluster-randomized prevalence survey. Mapping was undertaken as part of the study “Epidemiology of Yaws in the Solomon Islands and the Impact of a Trachoma Control Programme.” The full methodology and results of this survey are described elsewhere (5, 18). These data were collected in Western and Choiseul provinces of the Solomon Islands, September–October 2013. Demographic information is given in Web Table 1 in Web Appendix 1 (available at https://academic.oup.com/aje). The trachoma survey enrolled all participants regardless of age or sex and in these two provinces. This included a total of 5,838 individuals in 98 villages. In the yaws survey, children aged 5–14 years were enrolled, except in 1 cluster where the whole village was enrolled to allow more detailed data. This age group was selected for the overall sampling method because the majority of cases of yaws occur in this group, and interpretation of serology results is more difficult in adults, in whom positive results may reflect syphilis.

In each province, 25 clusters—consisting of villages or groups of villages of approximately equal size—were selected at random. In each cluster, a complete census of all households was obtained, and 30 households were selected using simple random sampling. In the selected households, children aged 5–14 years of age were invited to participate. Demographic data on age and sex were collected, along with clinical data on the presence or absence of skin or bone lesions consistent with primary or secondary yaws. Regardless of the presence of clinical signs of disease, a serum sample was collected from all children. Serum was tested at London School of Hygiene and Tropical Medicine with the *Treponema pallidum* particle agglutination assay (TPPA) (Mast Diagnostics, Merseyside, United Kingdom). Individuals with a positive TPPA had a quantitative rapid plasma reagin assay (RPR) (Deben Diagnostics, Ipswich, United Kingdom) performed. Individuals with clinical signs of yaws and both a positive TPPA and an RPR titer of ≥1:4 were considered to have active yaws. Individuals with both a positive TPPA and an RPR titer of ≥1:4 but without clinical signs of yaws were considered to have asymptomatic infection. Ethical approval for the survey was granted by the ethics committees of the Ministry of Health and Medical Services in the Solomon Islands (HRC 13/10) and London School of Hygiene and Tropical Medicine (6319 and 6358) in the United Kingdom.

### Model

Here we present the model used and the methods employed to fit it to the data. Further detail is given in Web Appendix 1 and Web Table 2, and code to simulate and fit the model is provided as Web Appendix 2. For the purposes of the model, we used the Ministry of Health Zones of the two provinces within which the survey had been conducted.

**Figure 1.**
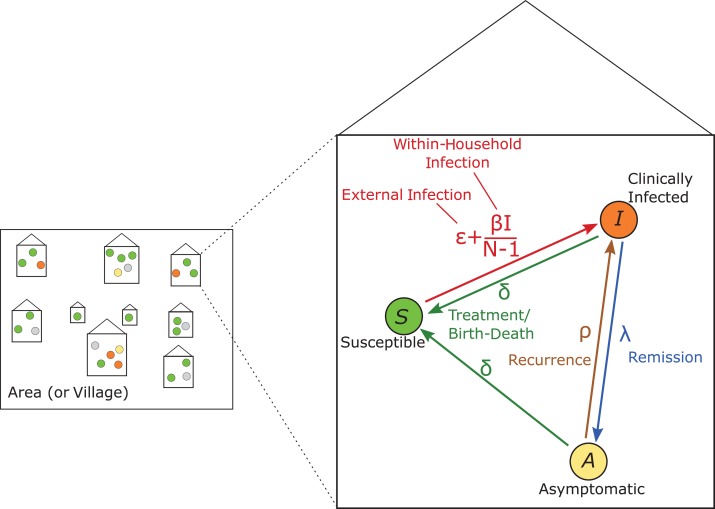
Schematic of the model structure for transmission of yaws. Each individual in the studied population is in a household of a different size, with the observed number of adults and children. Each individual is in one of the states in the model: susceptible (*S*, green); infected and showing clinical symptoms (*I*, orange); or infected and asymptomatic (*A*, yellow). The inset shows the life course that each individual can go through, from *S* to *I* to *A* at different rates. Parameters comprise: external infection, ε; within-household infection at a scaled rate, β; disease remission, λ; disease recurrence, ρ; and treatment/birth-death, δ. At equilibrium the population will have a particular distribution of the different states in households of different sizes.

To analyze the data, we used the household model summarized in Figure 1. Individuals were classified into 3 groups: susceptible (*S*, those without evidence of infection); asymptomatic (*A*, those that have the bacterial infection but do not display clinical signs); and infectious (*I*, those who show clinical signs of the disease). Only individuals with clinical signs (in the *I* category) can infect others, while asymptomatic individuals are not infectious. Within each household of size *N*, the rate of infection is split into two parts: ε and β. ε represents the force of infection that is external to the household, which may differ between areas but is the same for all households in a given area. For example, this could be transmission occurring between children at school. In the following analysis ε is assumed to be constant. In addition, each individual experiences a within-household infection rate (β) that depends on the proportion of other household members that are infectious.

Following an initial infectious period, skin lesions may heal spontaneously without treatment at a rate λ. Such individuals become asymptomatic (while remaining infected) with the bacterium (*A*) and, without treatment, clinical signs recur at a rate ρ. In addition, we represented treatment of the disease (or, equivalently birth and death events) by a rate δ from both the infectious and the asymptomatic compartments to the susceptible compartment. Because individuals cannot successfully clear the bacterium without treatment, there is no return to susceptibility without treatment.

### Analysis

We took the rate of recurrence of the disease to be ρ = 0.0167/month and the rate of disease remission to be λ = 0.185/month, consistent with expert opinion (19, 20), and fitted the remaining parameters in the model to the data using Markov Chain Monte Carlo (MCMC) methods to estimate the posterior distribution of model parameter values from the data. To find the household distributions of *I*, *S*, and *A* for each household size, we solved the master equation (also known as the Kolmogorov forward equation) at steady state (see Web Appendix 1), assuming each household was independent of all other households. To efficiently solve this system of linear equations, we converted it into a matrix system and added an additional line to represent the requirement that the probabilities of all possible states of the system must sum to unity, before solving this matrix system numerically. Markov Chain Monte Carlo thinned chains and histograms from those chains are shown in Web Figures 1 and 2.

To account for missing data (individuals who did not undergo serological testing, 13.7% of individuals in the data set) we conditioned our likelihood function on the statuses of these individuals and summed over all possible statuses. We used the posterior parameter value distributions to create a distribution of augmented data sets and, from these, estimated prevalences and statistics of interest.

For more details on the model fitting procedure, taking into account missing data, see Web Appendix 1.

## RESULTS

### Parameter values

Our fitted parameters are shown in Table 1, where each value given is the maximum of the posterior distribution, and the ranges given are the 95% credible intervals. Values of the external force of infection (ε) for different areas range from 0.0007 to 0.0078, while the within-household infection rate (β) and the treatment/birth-death rate (δ) are at least an order of magnitude greater in all areas considered, at 0.0516 and 0.0513, respectively, and ρ is 0.0165. Note that the values of ε and β are not directly comparable because the actual rate at which infection occurs also depends on the proportion of the rest of the household members who are infectious.

**Table 1.** Parameters for Different Areas Fitted Using Markov Chain Monte Carlo Methods, With Each Parameter Represented by the Maximum Posterior Value, Using Data From the Solomon Islands, 2013

Parameter	Area	MPV	95% CI
External force of infection (ε, per month)	Central Islands Western Province	0.0040	0.0024, 0.0065
	East New Georgia	0.0056	0.0034, 0.0091
	North Choiseul	0.0007	0.0003, 0.0016
	North West Choiseul	0.0024	0.0015, 0.0038
	Ranogga/Simbo	0.0076	0.0046, 0.0123
	Shortland Islands	0.0078	0.0041, 0.0132
	South Choiseul	0.0009	0.0004, 0.0017
	Vella La Vella	0.0059	0.0036, 0.0090
	West New Georgia	0.0043	0.0027, 0.0069
Within-household infection rate, β, per person, per month	All	0.0516	0.0240, 0.0848
Treatment/birth-death, δ, per month	All	0.0513	0.0360, 0.0707
Recurrence rate, ρ	All	0.0165	0.0136, 0.0190

Abbreviations: CI, credible interval; MPV, maximum posterior value.

### Assessing model fit

**Figure 2.**
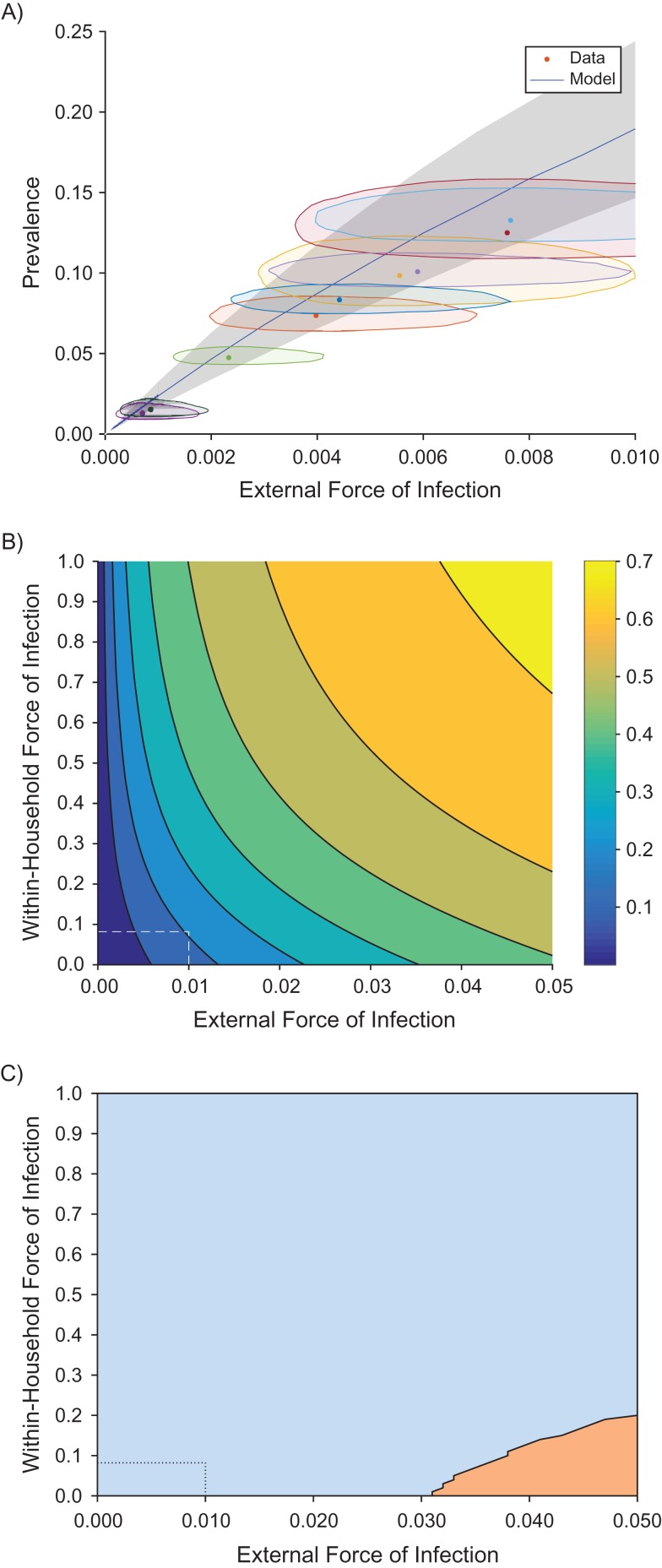
Model predictions of the prevalence of infection (clinical and asymptomatic), comparing data to prevalence predictions. A) Model predictions using the fitted value of β (blue line) with parameter uncertainty (gray area), compared to data (dots with uncertainty regions) from the Solomon Islands, 2013. The shaded area around each point is the 95% credible area, representing combined uncertainty in the estimation of the external force of infection (ε) and the imputed prevalence values (see Web Appendix 1). B) Prevalence predictions for varying within-house (β) and external (ε) infection rates. The colors show increasing prevalences from blue to yellow (see color bar). C) The region in which increasing ε leads to a greater increase in prevalence than increasing β.

To assess our model fit, we plotted the prevalence of infectious and asymptomatic individuals as the external force of infection (ε) changes (Figure 2A). Figure 2A shows the model prediction (blue line) for the fitted parameter values, with the 95% Bayesian credible interval in β (gray area). Each geographical area in the data set has a different fitted external infection rate (ε) and a different prevalence of infected individuals (*I* + *A*), which is plotted as points in Figure 2A. Individuals with unknown statuses were estimated during the analysis. The shaded area around each point is the 95% credible area, representing combined uncertainty in the estimation of the external force of infection (ε) and the imputed prevalence values (see Web Appendix 1).

**Figure 3.**
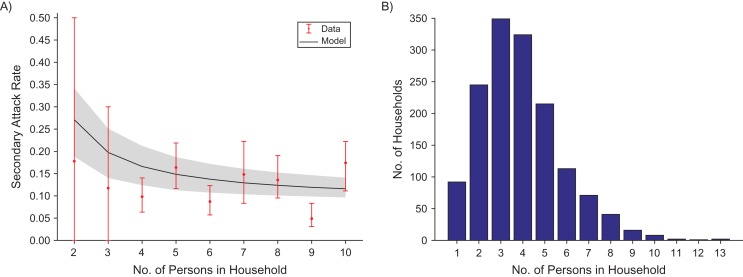
Model predictions of the secondary attack rate for yaws (A), taking the fitted value of β and ε (blue line) with parameter uncertainty (gray area) and comparing to data (dots) with error bars for different household sizes *N*, and the distribution of household sizes (B), using data from the Solomon Islands, 2013.

Our model assumed that a susceptible individual in a household with an infectious individual was less likely to become infected in a household with more people. To test this assumption, we calculated the mean secondary attack rate, which, for a household of size *N*, is found by considering 1 infectious individual in each household to be the “initial” case in the household (see, for example, House et al. (21)). The secondary attack rate for that household was then the proportion of household contacts of that initial case that were themselves infected (*I* + *A*). We plotted the mean of these values for each household size and compared this against the model predictions for this statistic. We found that the model fitted the data well (Figure 3A). We note that the large error bars for low household sizes arose because in a household of size 2, the status of one unknown individual will have a large effect on the secondary attack rate of that household. In addition, values of the secondary attack rate for large household sizes are also uncertain due to the low number of households at these sizes (Figure 3B).

### For realistic parameter values, decreasing external infection is more effective than decreasing within-household infection

Our model can be used to make predictions for how the prevalence of infected individuals (*I* + *A*) changes with changing external (ε) and within-household (β) infection rates (Figure 2B). In particular these predictions allow us to investigate the question, “should we focus on reducing within-household infection?” From Figure 2B we can see that in most of the considered parameter space, the prevalence increased more quickly if we moved parallel to the *x*-axis. To see this more directly, we plotted the areas in which the gradient with respect to the external force of infection, ε, was greater than that with respect to the within-household infection rate, β (Figure 2C). More explicitly, this figure reveals that, for realistic parameter values it is better to focus on reducing the prevalence of infection in the general area (i.e., ε), rather than trying to reduce infection within the household (i.e., β).

### Treating only cases and their household contacts left 70%–100% of asymptomatic individuals untreated

**Figure 4.**
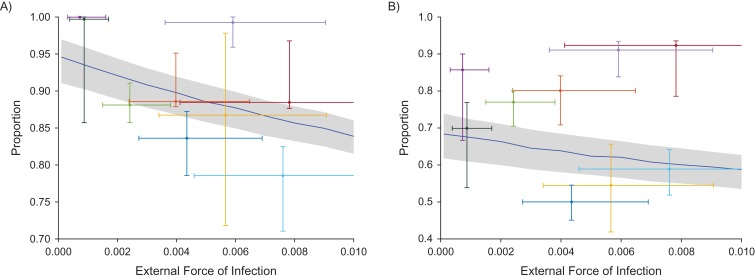
The people that treating cases and their household contacts for yaws misses as a fraction of all asymptomatic, infected people (A) and as a fraction of all infected (symptomatic or asymptomatic) people (B), using data from the Solomon Islands, 2013. Both plots give data points as colored dots (giving the maximum posterior density), with vertical and horizontal error bars representing the 95% credible intervals due to uncertainty in the fitted parameter, ε (the external force of infection), and in the proportion that would be missed (which is uncertain due to unknown individuals in the data set). Each plot has a blue line giving the model prediction and gray credible interval (due to uncertainty in parameter fitting). All plots vary the external force of infection (ε), with the equivalent prevalence (as found in Figure 2A) given in the upper *x*-axis. Full distributions of points can be found in Web Figure 3.

To investigate the effectiveness of treating only active cases and their household contacts, we assessed the proportion of asymptomatic, infected individuals who live in households with no active cases. These are individuals who would not receive treatment under the proposed scheme (using household contacts as a proxy for more general contacts). Thus if this proportion is sufficiently high, then we may conclude that treating only cases and their known contacts is unlikely to be an efficient method of eliminating the disease. We plotted this proportion as the external force of infection, and thus the prevalence, changed (Figure 4(A)). For each geographical area in the data set, we plotted the fitted external force of infection against the proportion of asymptomatic individuals who were untreated in that area. There is uncertainty in these values due to uncertainty in the fitted parameters and due to the missing data. Full distributions of points can be seen in Web Figure 3. Values from the data set ranged from approximately 70% up to 100% of asymptomatic individuals missed using this method. The model prediction for the mean value as the external force of infection varied is given by the blue line, with a gray area representing the credible interval. This prediction ranged from 83.7% to 93.9% and actually increased as the external force of infection decreased, corresponding to a decrease in prevalence.

### Treating only cases and their household contacts left 40%–95% of all infected individuals (asymptomatic clinical) untreated

We demonstrated above that the proposed scheme left a large proportion of asymptomatic, infected people untreated. We note, however, that the total number of infected individuals was lower at lower prevalences, so that the absolute number of untreated asymptomatic individuals may be lower at lower prevalences even though it is a higher proportion of those who are asymptomatic. In addition, our model assumed that the system is at steady state. Changing treatment campaigns moves the system out of steady state. Thus, while this is suggestive of outcomes after prolonged TTT, we are not directly predicting the results of reducing prevalence in this way.

It could be argued that because the absolute number of asymptomatic individuals was lower at lower prevalences, then perhaps control is still possible while still missing a large proportion of asymptomatic infections. However, even assuming that every clinical infection is correctly identified and included in the scheme, the proportion of infected (symptomatic or asymptomatic) individuals who are missed under this scheme was 40%–95% (Figure 4B). Model predictions for the mean proportion of infected individuals who are missed varied between 61.7% and 68.8% and, again, are higher at lower prevalences. There also did not appear to be a strong correlation between prevalence and proportion missed, and switching to treating cases and contacts for realistic parameter values would still miss around 65% of infected (symptomatic or asymptomatic) individuals.

## DISCUSSION

To our knowledge, this is the first study to attempt to explore the role of both within-household and between-household transmission of yaws, and provides important information to guide yaws eradication efforts. Relapses of asymptomatic, infected individuals are a major driver of reemergence of yaws following initial control efforts (7), and obtaining adequate coverage of these individuals is therefore vital if yaws eradication is to be a success. There is currently limited understanding of the spatial and social interactions between clinical and asymptomatic cases of yaws in the community. A key finding of this study was that treatment of household contacts appears to provide only limited coverage of asymptomatic, infected individuals. Currently, the Morges strategy defines a contact as a person who has close and frequent contact with the infected person and is a member of the household, a classmate, or a close playmate as identified by the contact (2), but this definition is not based on any empirical data. While we have not directly addressed schools as sources of transmission, our data suggest that a broader definition of contacts may be necessary to achieve high coverage of asymptomatic, infected cases.

Given the need to achieve high coverage of asymptomatic, infected individuals, the limitations of household contact tracing, and the relatively fixed costs of returning to communities to conduct follow-up surveys, it may be more efficient and cost-effective to conduct multiple rounds of TCT rather than an initial round followed by TTT. Such an approach would ensure high coverage of asymptomatic, infected people and might allow alignment of yaws eradication efforts with other neglected tropical disease programs based on annual or semiannual TCT, which should improve cost-effectiveness. Trials to assess the potential of these alternative TCT strategies should be considered to facilitate scale-up of eradication efforts.

In a previous study, an attempt was made to define coverage thresholds at which interruption of transmission might be achieved (20), concluding that even at low estimates of *R*_0_, 8 rounds of treatment at 80% coverage would be required to have an 80% chance of eradication. However, this did not explicitly address the question of how likely specific mass treatment strategies were to achieve these thresholds. The current model adds to the earlier data by explicitly examining the likelihood of achieving adequate coverage with a particular strategy based on treatment of cases and their household contacts. Our data demonstrated that coverage using this strategy would be low and would not reach the thresholds suggested by earlier modeling work.

This study has a number of limitations. First, data were not available for every individual in each house. Adults were deliberately not included in the original survey from which this data set is derived (5) because serological tests cannot distinguish syphilis infection from yaws (22). Second, we did not collect data on other sites of mixing, such as schools or churches, which might act as foci of transmission, and this limits our ability to examine broader definitions of contact tracing and treatment. In particular our modeling considers only household contacts, which may or may not be the major route of infection transmission. Third, data were collected at a single point in time, which limits our ability to directly examine how within- and between-household forces of infection vary following intervention. Fourth, the model was fitted at steady state, which may not adequately represent the yaws situation in the pre-TCT communities surveyed. Although we assessed the proportion of asymptomatic infections that would be missed by the Morges strategy, we have not conducted a full dynamic prediction of the potential impact of household-level tracing. Despite these limitations, we believe this is the first study to explore household transmission of yaws and provides important insights to guide programmatic scale-up of yaws eradication efforts.

Modeling has provided important insights into optimal approaches for many other neglected tropical disease programs (23, 24), but few models have been produced to date to guide yaws eradication efforts. In this study we have demonstrated that a household model can provide important insights into yaws transmission at the community level and inform program strategies.

Improving the approach to TTT is a priority for yaws eradication programs. Longitudinal epidemiologic studies collecting detailed data on potential foci of transmission will allow us to improve our understanding of yaws dynamics at the community level, improve on our current household model, and guide better targeted interventions. It would be of value to understand how these transmission foci change in the context of programmatic implementation and whether different methods for contact tracing are appropriate as programs progress. Alongside traditional epidemiologic and modeling approaches, next-generation sequencing shows significant promise as a tool to understand yaws transmission at the molecular level. Integrated longitudinal studies using these tools together would provide unparalleled insights into yaws transmission and allow an optimized approach to the detection of both active and asymptomatic infections. Such studies should be a priority.

## Supplementary Material

Web Material

## Abbreviations

RPR: rapid plasma reagin assay
TCT: total community treatment
TPPA: *Treponema pallidum* particle agglutination assay
TTT: total targeted treatment
