# Supplementary material for: Targeted Treatment of Yaws With Household Contact Tracing: How Much Do We Miss?
Source: Am J Epidemiol. 2017 Nov 13;187(4):837–44. doi: 10.1093/aje/kwx305 (PMC5888927; doi:10.1093/aje/kwx305)
Supplement: Web Material [file kwx305dysonwebmaterialfinal.zip › kwx305 Dyson Web Material Final/kwx305 Dyson Web Appendix 1.pdf]

TARGETED TREATMENT OF YAWS WITH HOUSEHOLD CONTACT TRACING: HOW MUCH DO WE MISS: WEB APPENDIX 1

Many of the techniques presented here are standard methods. We reproduce them here for convenience.

	N	Male	Female	Age (Median - IQR)
Health Zone A	149	71 (47.65%)	78 (52.355)	9 (7-11)
Health Zone B*	306	135 (44.12%)	171 (55.88%)	14 (9-36)
Health Zone C	111	66 (59.46%)	45 (40.54%)	9 (6-12)
Health Zone D	297	162 (54.55%)	135 (45.45%)	9 (7-11)
Health Zone E	130	70 (53.85%)	60 (46.15%)	9 (7-11)
Health Zone F	70	32 (45.71%)	38 (54.29%)	9 (7-12)
Health Zone G	174	86 (49.43%)	88 (50.57%)	9 (6-11)
Health Zone H	171	82 (47.95%)	89 (52.05%)	9 (7-12)
Health Zone I	237	115 (48.52%)	122 (51.48%)	9 (7-12)

Web Table 1: Demographic information

*In this cluster only the whole population was enrolled irrespective of age to allow more detailed analysis

Model definition

The model is given in Figure 1 of the main text. For a household of size N, the model is defined by the state space (S, I, A), taking integer values from 0 to N. The events and transitions rates are given in Web Table 2. We solve the model at steady state in the usual way, described below.

Event	Transition	Rate
Internal infection	$(S, I, A) \to (S-1, I+1, A)$	$\frac{\beta I}{N-1}$
External infection	$(S, I, A) \to (S-1, I+1, A)$	ϵ
Treatment / birth-death of infectives	$(S, I, A) \to (S+1, I-1, A)$	δ
Treatment / birth-death of asymptomatics	$(S, I, A) \to (S+1, I-1, A)$	δ
Recurrence of clinical infection	$(S, I, A) \to (S, I+1, A-1)$	ρ
Remission of clinical infection	$(S, I, A) \to (S, I-1, A+1)$	λ

Web Table 2: Transition probability fluxes for the model

Solving the household model at steady state

Our model is given by Figure 1 in the main text, and can be represented by the following master equation for the probability of a household of size N containing S susceptibles, I infectious and A asymptomatics:

$$\begin{aligned}\frac{\partial P^N_{S,I,A}}{\partial t} = &-\left(\left(\epsilon + \frac{\beta I}{N-1}\right) S + \delta(A+I) + \rho A + \lambda I\right) P^N_{S,I,A} + \left(\epsilon + \frac{\beta(I-1)}{N-1}\right)(S+1)P^N_{S+1,I-1,A} \\ &+ \delta(A+1)P^N_{S-1,I,A+1} + \delta(I+1)P^N_{S-1,I+1,A} + \rho(A+1)P^N_{S,I-1,A+1} + \lambda(I+1)P^N_{S,I+1,A-1},\end{aligned} \quad (1)$$

where $P^N_{S,I,A} = 0$ if $S < 0$, $I < 0$, $A < 0$ or $S + I + A \neq N$, and reactions are not included when they would lead to this situation. Noting that $S + I + A = N$, reduces this to a two-dimensional system, which we solve at steady state by setting the right hand side equal to zero and solving for $P_{S,I,A}$ for all possible values of S, I and A. In principle

for any given value of N it is possible to find an analytical solution, but in practice we solve the system numerically by converting it into the equivalent matrix equation:

$$M(\beta, \delta, \epsilon, \rho, \lambda, N)\mathbf{P}^N = \mathbf{0}, \tag{2}$$

where $\mathbf{P}^N$ is the vector of $P^N_{S,I,A}$ for all possible values of S, I and A for a household of size N, and $\mathbf{0}$ is the vector of zeros, the same size as $\mathbf{P}^N$. $M(\beta, \delta, \epsilon, \rho, \lambda, N)$ is a matrix representing the coefficients in equation (1). This set of equations does not fully define $\mathbf{P}^N$, since the matrix $M(\beta\delta, \epsilon, \rho, \lambda, N)$ is not of full rank. To overcome this we add an extra row of ones to the bottom of A and an extra 1 to the bottom of $\mathbf{0}$, representing the condition that $\sum_{S,I,A} P^N_{S,I,A} = 1$. This enables us to (numerically) invert the matrix and determine $P^N_{S,I,A}$.

Obtaining the likelihood function while accounting for missing data

The likelihood function is defined as the probability of observing our data, x, given some parameter set, θ. This can be calculated for each house in the dataset (that has no missing data) as $P^N_{S,I,A}$, for the values of S, I, A and N in the dataset. For houses that contain individuals with missing data, we condition on the value of those missing individuals. So we take the likelihood for that household to be

$$\pi_h(\theta) = P(x|\theta) = \sum_U P(x, U|\theta), \tag{3}$$

where U is the unknown values and the sum is taken over all possible states the unknown values could take. For a given value of U, $P(x, U|\theta)$ is simply the relevant value of $P^N_{S,I,A}$ (i.e. the number of S, I, and A given by x with U). To calculate the total likelihood, we assume all houses are independent and take

$$\pi(\theta) = \prod_h \pi_h(\theta). \tag{4}$$

In fact, to avoid numerical problems with very small values of π, we use the log likelihood, $log(\pi(\theta)) = \sum_h log(\pi_h(\theta))$.

Metropolis Hastings algorithm

We fit our model using a Metropolis Hastings implementation of Markov Chain Monte Carlo (MCMC). The algorithm consists of the steps below:

1. Propose a new set of parameters;
2. Check the parameters lie within the domain (otherwise reject the parameter set);
3. Accept with probability $\min(\exp[log(\pi(\bar{\theta})) - log(\pi(\theta)))]\frac{p_\rho(\bar{\rho})}{p_\rho(\rho)}$,1);
4. If $\bar{\theta}$ is accepted then set $\theta = \bar{\theta}$.
5. Record θ as one step in the chain
6. If the chain is long enough then stop, otherwise return to 1

This procedure gives us a chain of parameter sets.

For all parameters except ρ, the proposal distribution is taken to be $\bar{\theta} = \theta + \mathbf{R} \cdot \mathcal{N}(0, 1)$, where $\mathbf{R}$ is given below. For ρ we take the prior distribution p_ρ, to the normal distribution with mean 0.0067 and standard deviation 0.00067, and we take this to be our proposal distribution for that parameter.

We run 16 simultaneous chains, and for each chain we initiate the algorithm as follows. Initial parameters are taken uniformly at random between 0 and 0.1, (except for ρ, which is initialised to be 0.0167), with a different initialisation for each chain. We then run 50,000 iterations with the standard deviation of the proposal distribution taken to be 0.001. We then run an additional 50,000 iterations with the standard deviation of the proposal distribution given by $\mathbf{R} = (0.0003, 0.0006, 0.0001, 0.0002, 0.0006, 0.0006, 0.0001, 0.0004, 0.0003, 0.0067, 0.0067)$ (set by hand to ensure good mixing of the chain). We discard these 100,000 iterations as burn-in and run a further 100,000 iterations with the standard deviation of $\mathbf{R}$. We use flat priors, uniform on $(0, \infty)$ for all parameters except for ρ.

We thin our chain by an interval of 100, so that we keep only every 100th value, to reduce chain autocorrelation. Since we take 1600,000 iterations (not including burnin), that gives us a final chain of 16,000 values from which we take the maximum of our posterior distribution, the 95% credible intervals and areas, and other statistics. The chains are concatenated together and shown in Web Figure 1. Posterior histograms generated by these chains are shown in Web Figure 2.

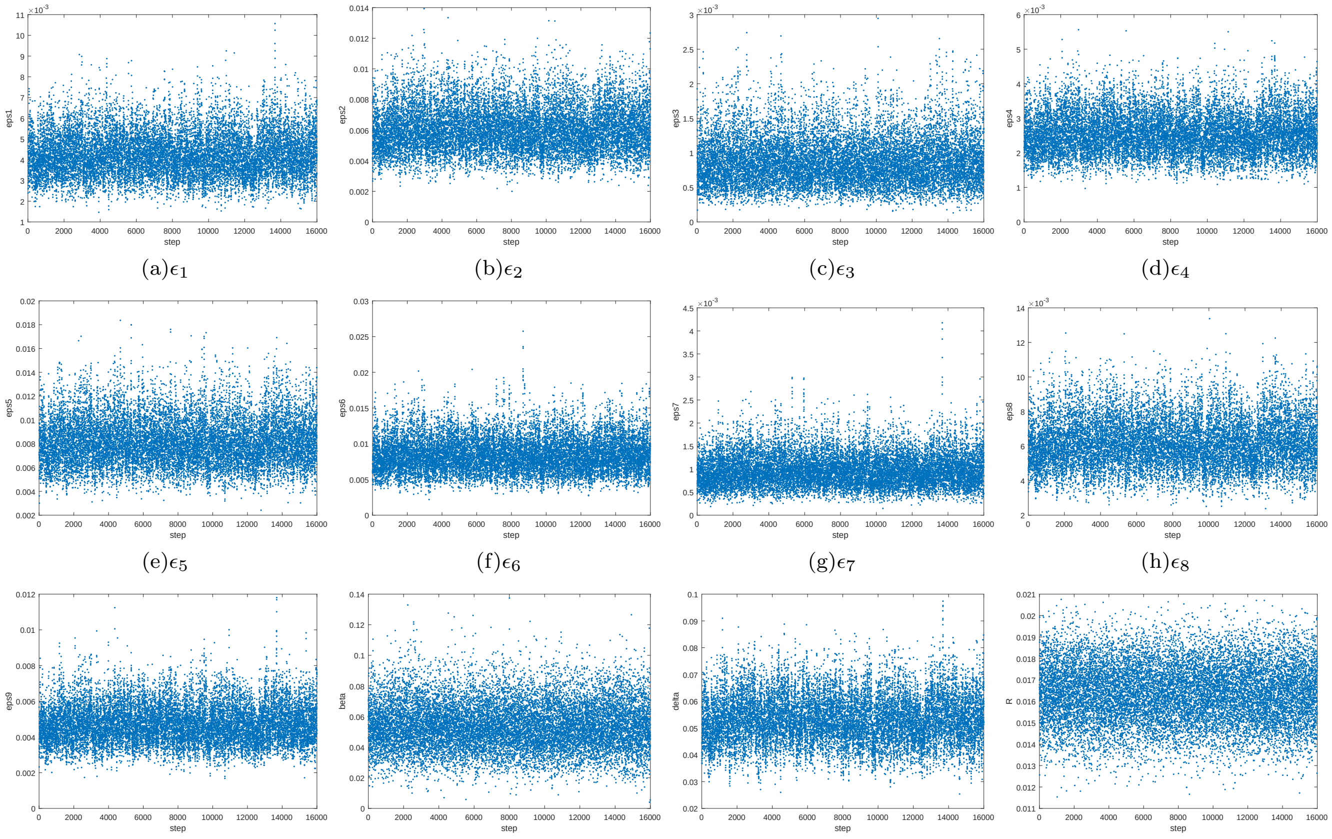

(a)ϵ_1 (b)ϵ_2 (c)ϵ_3 (d)ϵ_4 (e)ϵ_5 (f)ϵ_6 (g)ϵ_7 (h)ϵ_8 (i)ϵ_9 (j)β (k)δ (l)ρ

Web Figure 1: Thinned chains generated by MCMC

Assessing chain convergence and effective sample size

We calculate the effective sample size (ESS) for our MCMC chains using multiESS from the R package mcmcse. This is calculated as

$$\text{ESS} = n\frac{|\Lambda|^{1/p}}{|\Sigma|^{1/p}}, \tag{5}$$

where Λ is the sample covariance matrix for g and Σ is an estimate of the Monte Carlo standard error for g. In our case g is the identity, which is the estimate of the mean of the target density. Our chains have a total ESS of 10062.39. This is above the minimum ESS for a chain with 12 variables, calculating at 95% confidence, with a tolerance of 0.05, which is calculated to be 8826 by minESS from the same R package. This is calculated as

$$\text{minESS} = \frac{2^{2/p}\pi}{(p\Gamma(p/2))^{2/p}}\frac{\chi^2_{1-\alpha,p}}{\epsilon^2}, \tag{6}$$

where p is the dimension of the estimation problem, α is the confidence level and ϵ is the tolerance level.

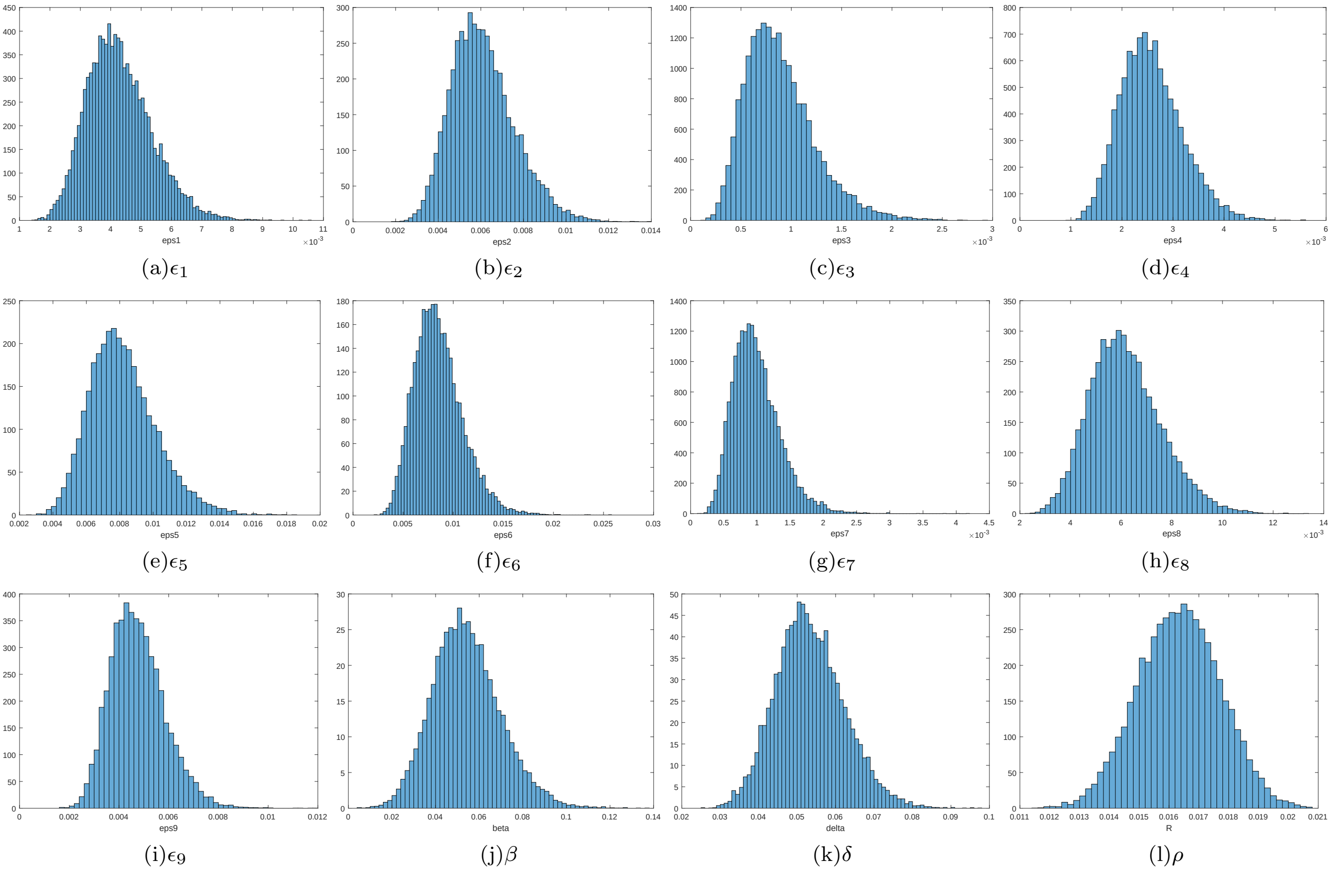

(a)ϵ_1 (b)ϵ_2 (c)ϵ_3 (d)ϵ_4

(e)ϵ_5 (f)ϵ_6 (g)ϵ_7 (h)ϵ_8

(i)ϵ_9 (j)β (k)δ (l)ρ

Web Figure 2: Histograms generated by thinned MCMC chains

To assess chain convergence we use the Gelman-Rubin-Brooks multivariate potential scale reduction factor MCMC convergence diagnostic [1]. This generalises the Gelmin-Rubin method to consider more than one parameter simultaneously. The method uses multiple chains and considers the difference between the within-chain variance and the between-chain variance. If the chains have all reached steady state then we would expect these two variances to be the same, and for their ratio to be close to one. We obtain a value of $R = 1.02$ and conclude that our chains have converged.

Estimating statistics from the MCMC chain

We can use the MCMC chain calculated above to estimate other statistics that depend on the value of the unknown data from the MCMC chain, by drawing parameter sets from the MCMC chain and using these, together with the known data, to determine the probability distribution of the unknown data. So for each parameter set we draw from the MCMC chain, we then find the probability distribution of the unknown values and draw the unknown values from that and calculate the statistics we require. This gives us a posterior probability distribution for the statistics.

To find the probability distribution for the unknown values, we consider a household of size N with I^*, S^* and A^* observed infectious, susceptibles and asymptomatics and U people of unknown status. In this household the

probability that there are I, S and A individuals, given that we observe I^*, S^* and A^* is:

$$P(I,S,A|I^*,S^*,A^*) = \frac{P(I,S,A \cap I^*,S^*,A^*)}{P(I^*,S^*,A^*)}, \tag{7}$$

$$= \frac{P(I,S,A)P(\text{choose } I-I^*, S-S^*, A-A^* \text{ to be unknown})}{P(I^*,S^*,A^*)}, \tag{8}$$

$$= \frac{P(I,S,A)MultiHyperGeom([I,S,A],[I-I^*,S-S^*,A-A^*])}{P(I^*,S^*,A^*)}, \tag{9}$$

where $MultiHyperGeom([I,S,A],[\bar{I},\bar{S},\bar{A}])$ is the multivariate hypergeometric distribution, which gives the probability of selecting $[\bar{I},\bar{S},\bar{A}]$ at random from a population of $[I,S,A]$. In practise we don't need to find $P(I^*,S^*,A^*)$ because we can just normalise so that $\sum_{I,S,A} P(I,S,A|I^*,S^*,A^*) = 1$

Mean SAR (Figure 3(a))

So for each house with missing data we draw multiple times from the distribution given by $P(I,S,A|I^*,S^*,A^*)$ and calculate the prevalence of $I+A$, the proportion of asymptomatics that live in houses without infectious people (and hence would be untreated), the proportion of infections (I+A) that would be untreated, and the mean secondary attack rate (SAR).

We define the mean secondary attack rate (SAR) for a household of size N to be the proportion of all household contacts of infected individuals who are themselves infected or asymptomatic:

$$SAR_N = \frac{1}{H_N}\sum \frac{(I(\text{household}) + A(\text{household}) - 1)}{N(\text{household}) - 1}, \tag{10}$$

where H_N is the number of households of size N, and the summation is taken over all households of size N with at least one infective individual.

Calculating 95% credible intervals and credible areas

For each parameter we report the mode of the posterior distribution, and calculate the smallest interval in which 95% of the data lies (the 95% credible interval). When plotting the prevalence of infection against the external rate of infection (ϵ) there is uncertainty in the value of ϵ, but there is also uncertainty in the prevalence, due to people of unknown status. Since the imputed prevalence will depend on the value of ϵ, we calculate credible areas rather than two independent credible intervals. For the credible area we first use kernel density smoothing with a normal kernel (using the function ksdensity in Matlab)[2] to smooth the output before calculating the 95% credible area as the smallest contour within which 95% of the output lies.

Model predictions

We use the solution to equation (2) to calculate the mean values of quantities that we wish to predict for each value of the household size, N. We then weight these predictions using the distribution of household sizes found in the dataset. The prevalence of infection ($I+A$) is given by

$$\langle \text{prevalence} \rangle = \sum_{N,I,A}\sum_{S}(N-S)P^N_{S,I,A}H_N, \tag{11}$$

where H_N is the fraction of households in the dataset that are of size N. Similarly the secondary attack rate is given by

$$\langle SAR \rangle = \sum_{N,I,A}\sum_{S=0}^{N-2}(N-S-1)\frac{P^N_{S,I,A}}{\sum_{S=0}^{N-2}\sum_{I,A}P^N_{S,I,A}}H_N, \tag{12}$$

the proportion of asymptomatics that are in households with no clinical infections (*i.e.* $A > 0$ and $I = 0$) is given by

$$\langle A > 0, I = 0 \rangle = \sum_{N,S} \sum_{A=1}^{N-1} A P^{N}_{S,I=0,A} H_N, \tag{13}$$

and the proportion of infections $(A + I)$ that are in households with no clinical infections

$$\langle \text{proportion of infections} \rangle \approx \frac{\langle A > 0, I = 0 \rangle}{\langle A + I \rangle}, \tag{14}$$

$$= \frac{\sum_{N,S} \sum_{A=1}^{N-1} A P^{N}_{S,I=0,A} H_N}{\sum_{N,I} \sum_{S=0}^{N} (N - S) P^{N}_{S,I,A} H_N}, \tag{15}$$

since $N - S = I + A$.

Determining the region in which reducing ϵ is more effective (Figure 2(c))

We determine the region in which reducing ϵ is more effective by taking the numerical derivative of the prevalence of infection with respect to β and with respect to ϵ and determining which is larger. That is, we compare the reduction in prevalence due to reducing β by a small amount, to the reduction in prevalence due to reducing ϵ by a small amount.

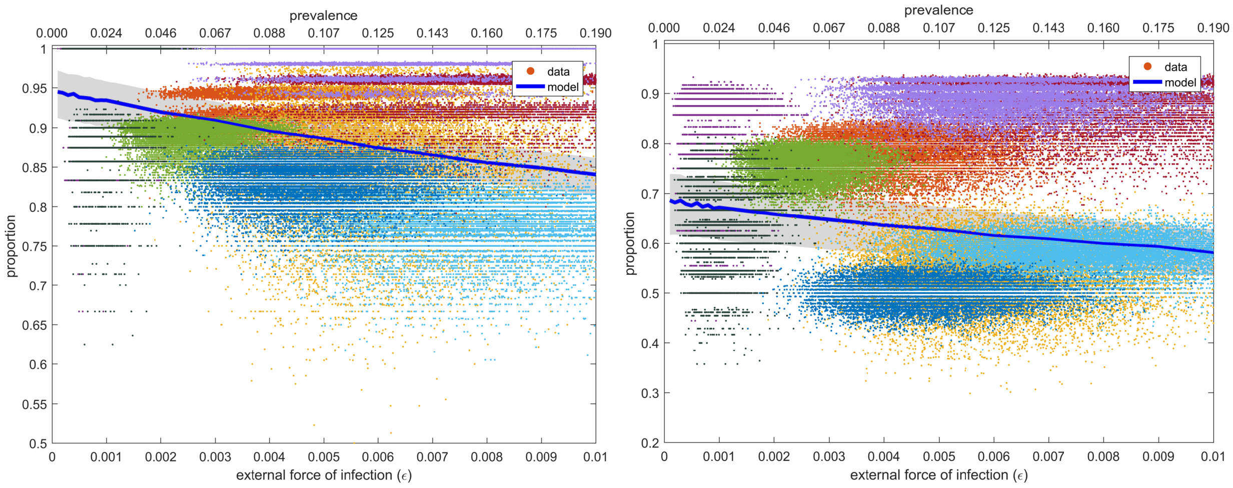

(a)Proportion of asymptomatics that would be untreated (b)Proportion of infections $(A + I)$ that would be untreated

Web Figure 3: An extended version of Figure 4 in the main text. The people that treating cases and their household contacts misses (a) as a fraction of all asymptomatically infected people; and (b) as a fraction of all infected (symptomatically or asymptomatically) people. Both plots give data from each area as a cloud of points representing the uncertainty in the values of both the external force of infection (ϵ), and the proportions of infections that would be missed (which is uncertain due to unknown individuals in the dataset). Each plot has a blue line giving the model prediction and grey credible interval (due to uncertainty in parameter fitting). All plots vary the external force of infection (ϵ), with the equivalent prevalence given in the upper x-axis.

[1] S. P. Brooks, G. O. Roberts, Convergence assessment techniques for Markov chain Monte Carlo, Statistics and Computing 8 (4) (1998) 319–335. `\path{doi:10.1023/A:1008820505350}`.

[2] A. W. Bowman, A. Azzalini., Applied Smoothing Techniques for Data Analysis, Oxford University Press, 1997.
